# NemaPath: online exploration of KEGG-based metabolic pathways for nematodes

**DOI:** 10.1186/1471-2164-9-525

**Published:** 2008-11-04

**Authors:** Todd Wylie, John Martin, Sahar Abubucker, Yong Yin, David Messina, Zhengyuan Wang, James P McCarter, Makedonka Mitreva

**Affiliations:** 1 The Genome Center at Washington University School of Medicine, St. Louis, MO 63108, USA; 2 Divergence, Inc., 892 North Warson Road, St. Louis, MO 63141, USA

## Abstract

**Background:**

Nematode.net  is a web-accessible resource for investigating gene sequences from parasitic and free-living nematode genomes. Beyond the well-characterized model nematode *C. elegans*, over 500,000 expressed sequence tags (ESTs) and nearly 600,000 genome survey sequences (GSSs) have been generated from 36 nematode species as part of the Parasitic Nematode Genomics Program undertaken by the Genome Center at Washington University School of Medicine. However, these sequencing data are not present in most publicly available protein databases, which only include sequences in Swiss-Prot. Swiss-Prot, in turn, relies on GenBank/Embl/DDJP for predicted proteins from complete genomes or full-length proteins.

**Description:**

Here we present the NemaPath pathway server, a web-based pathway-level visualization tool for navigating putative metabolic pathways for over 30 nematode species, including 27 parasites. The NemaPath approach consists of two parts: 1) a backend tool to align and evaluate nematode genomic sequences (curated EST contigs) against the annotated Kyoto Encyclopedia of Genes and Genomes (KEGG) protein database; 2) a web viewing application that displays annotated KEGG pathway maps based on desired confidence levels of primary sequence similarity as defined by a user. NemaPath also provides cross-referenced access to nematode genome information provided by other tools available on Nematode.net, including: detailed NemaGene EST cluster information; putative translations; GBrowse EST cluster views; links from nematode data to external databases for corresponding synonymous *C. elegans *counterparts, subject matches in KEGG's gene database, and also KEGG Ontology (KO) identification.

**Conclusion:**

The NemaPath server hosts metabolic pathway mappings for 30 nematode species and is available on the World Wide Web at . The nematode source sequences used for the metabolic pathway mappings are available via FTP , as provided by the Genome Center at Washington University School of Medicine.

## Background

### Phylum Nematoda and Nematode Genomics

Nematodes or roundworms are one of the most common phyla of animals, with over 20,000 different described species [1], ubiquitous in freshwater, marine, and terrestrial environments. They have remarkable life-styles both in free-living and parasitic variants, having the ability to adapt to challenging environments or to invade multiple hosts, respectively. Parasitic nematodes of humans cause sub-clinical and clinical diseases of major socio-economic importance globally as ~3 billion people are infected [2]. Financial losses caused by parasites to agriculture (domesticated animals and crops) have a major impact on farm profitability, exacerbating global food shortage situations (e.g., plant parasitic nematodes are responsible for $80 billion in annual crop damage [3]). Nematodes have been studied extensively due to their agricultural and medical importance; nematode sequencing data have increased at a rapid rate over the past decade. As of the beginning of 2008, there are over 500,000 Expressed Sequence Tags (ESTs) in the dbEST division of GenBank originating from over 40 non-*Caenorhabditis *nematode species [4], and 32 genomic projects are completed or underway [5].

We expect that complete annotated genomes are approximately 3–4 years away. Therefore, to empower the broader scientific community the available EST and GSS sequences from parasitic nematodes require organization in a functional context, a need underscored by their absent from the majority of publicly available protein databases such as Pfam [6] and Kyoto Encyclopedia of Genes and Genomes (KEGG) [7]. These protein databases incorporate only sequences found in Swiss-Prot [8], and Swiss-Prot in turn relies on GenBank/EMBL/DDJP for predicted proteins from full genomes or full-length proteins. Hence, nematode-originated EST and GSS sequence data can only be informative when organized and presented in an easily accessible, systematic way that can be explored by the scientific community.

Currently, there are four comprehensive web-based databases available providing tools for exploring nematode sequences: two model-species-specific databases and two others encompassing parasitic and free-living nematode sequences. The model-specific databases include WormBase [9], a model organism database for *Caenorhabditis elegans *and other related *Caenorhabditis *species, and Pristionchus.org [10], a resource dedicated to the major satellite organism *Pristionchus pacificus *used in studying evolutionary developmental biology. NEMBASE3 [11] and Nematode.net [12] are databases concerned with the aggregation and navigation of sequencing data derived from multiple free-living and parasitic nematode species.

### Nematode Pathway Visualization and Comparison

Here we present NemaPath (made available as a component of Nematode.net) that allows for systematic study of nematode transcriptomes via enzyme pathway associations. NemaPath compares relatively refined nucleotide sequences (e.g., full-length cDNAs, clustered ESTs, RefSeq) to the KEGG genes database of curated protein sequences. Sequences in the KEGG database have known, annotated Enzyme Commission (EC) [13] system number associations. By aligning query sequence against annotated sequence we may assign putative function by EC number association. The software discards KEGG database entries that do not have associated EC numbers, and therefore only displays metabolic pathways. As our software makes no assumptions based on prior biological or biochemical knowledge, the end-user is able to navigate the full set of returned alignment summaries (strength of hit as E-value and bit score, subject accession numbers, etc.) from initial exploratory query sequences.

To eliminate data redundancy, ESTs have been assembled by identity into NemaGene contigs and further organized into clusters. ESTs within a contig derive from nearly identical transcripts, whereas contigs within a cluster may represent splice isoforms of a gene or transcripts from multi-gene families with extremely high sequence identity [14]. NemaPath associations are done on the contig level using NemaGene contig builds as querying sequences or full-length genes resulting from genome projects (e.g., *C. elegans*, *Brugia malayi*, *Ancylostoma caninum *and *Ascaris suum*). Cluster sequence reports may be viewed online by reverse lookup using constituent contig IDs from NemaPath's pathway map hit table or by viewing the cluster in Nematode.net's implementation of the GBrowse [15] genome viewer.

Finally, summaries of mappings are provided as extendable tree-views, which organize mappings by identified EC numbers. The summary of pathway mappings when coupled with statistical tools [16] can provide the scientific community with a solid platform for comparative metabolomics in the Phylum Nematoda.

Although other excellent KEGG pathway mapping software platforms exist for EST-related data – such as ESTExplorer [17] and PathwayExplorer [18] – the main aim of the NemaPath database is to provide pre-compiled pan-phylum comparative metabolomics for an important and oft-studied group of parasites, as well as cross-integration with other nematode information (e.g., WormBase). As such, new sequence data are added as they become publicly available without effort at the end-user level. Many of the nematode-centric KEGG views we provide are unique unto NemaPath.

## Construction and Content

**Figure 1:**
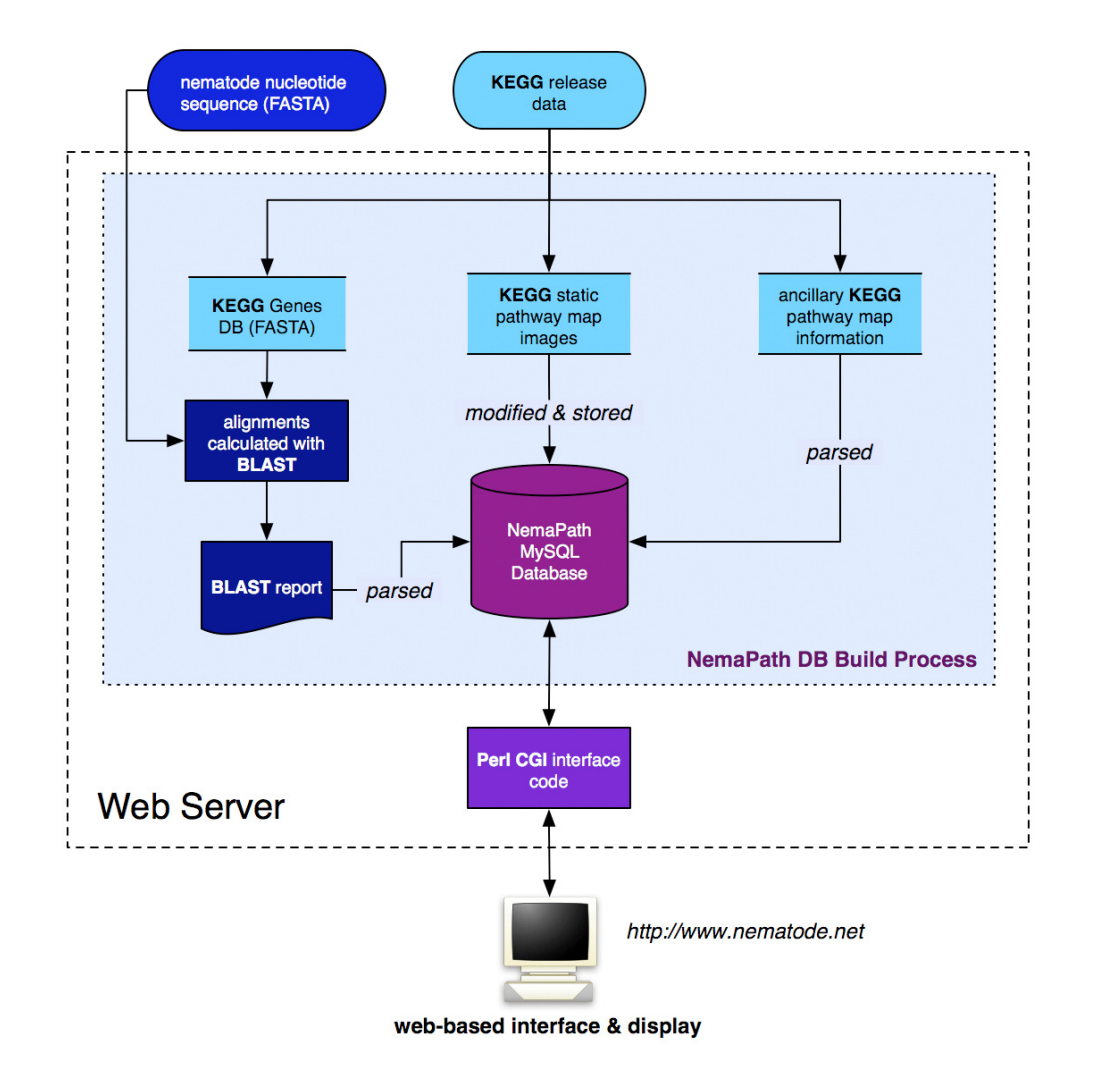
**NemaPath Pipeline Work Flow**. The general workflow of the NemaPath application. FASTA sequences feed the pipeline in the form of multi-species gene sequences and multi-nematode sequences, as provided by KEGG and Nematode.net, respectively. KEGG release data (map images, bitmap coordinates, node information) are correlated and placed in the database. KEGG gene alignments are performed and stored. Users may interrogate the database dynamically and display results through the World Wide Web.

The web-based NemaPath application (see Figure 1 for workflow) consists of two distinct components: 1) a server-side application to align and evaluate nematode transcript and gene sequences against the manually annotated KEGG genes database; 2) a browser accessible pathway viewing application for displaying associations. Both components are written in Perl [19], the interpreted procedural programming language. A MySQL [20] relational database holds shared data and acts as an intermediate between the two components.

### Alignment Association to KEGG

The initial step in the NemaPath pipeline involves analyzing clustered ESTs in context of the latest version of KEGG's high-quality genomes including manual assignment of orthologies. NemaGene EST contigs [[21], *Materials and Methods*] are a value-added effort, refinements include: 1) grouping of the ESTs into contigs based on sequence similarity; 2) elimination of chimeric ESTs; 3) accommodation of multiple splice-isoforms; 4) persistence of cluster names when new ESTs are added. For each contig with multiple EST members, the consensus sequence is longer and of higher quality than each stand-alone EST read. The KEGG genes file (used as a database against which we BLAST our queries) is comprised of concatenated FASTA protein sequences from all of the species in the KEGG genes database. As of this writing, the KEGG genes file can be retrieved for academic use by download via FTP from KEGG . Each FASTA entry contains metadata in the header line: species gene name, protein matched, EC id, and KEGG Orthology (KO) information. As the metadata represents information arrived at by KEGG's annotation process, we can make inferred relationships by identity using our nematode sequences.

WU-BLAST [22] alignments are performed in an automated fashion by a perl application called KEGGscan. KEGGscan reports compile BLAST results in a tab-delimited format, including E-value and bit score for the alignment, as well as the metadata information pulled from the KEGG subject's header line. KEGGscan reports are loaded into the NemaPath database during every NemaPath build; plaintext report file for each species/build can be accessed through our web site .

### Pathway-Association Navigation

KEGGscan report information is cross-referenced with corresponding KEGG pathway image map information in the NemaPath database. EC ids have representative nodes within KEGG-supplied pathway bitmap images. Associations between NemaGene contig sequence and KEGG genes are pre-compiled on a per build basis for each NemaPath release. However, a user has the ability to narrow or broaden the pool of viewable matches by supplying a threshold for E-value, which is a statistical value of the quality of the BLAST alignment. To aid the user in this endeavor, NemaPath supplies an E-value hit distribution graph for each nematode species.

**Figure 2:**
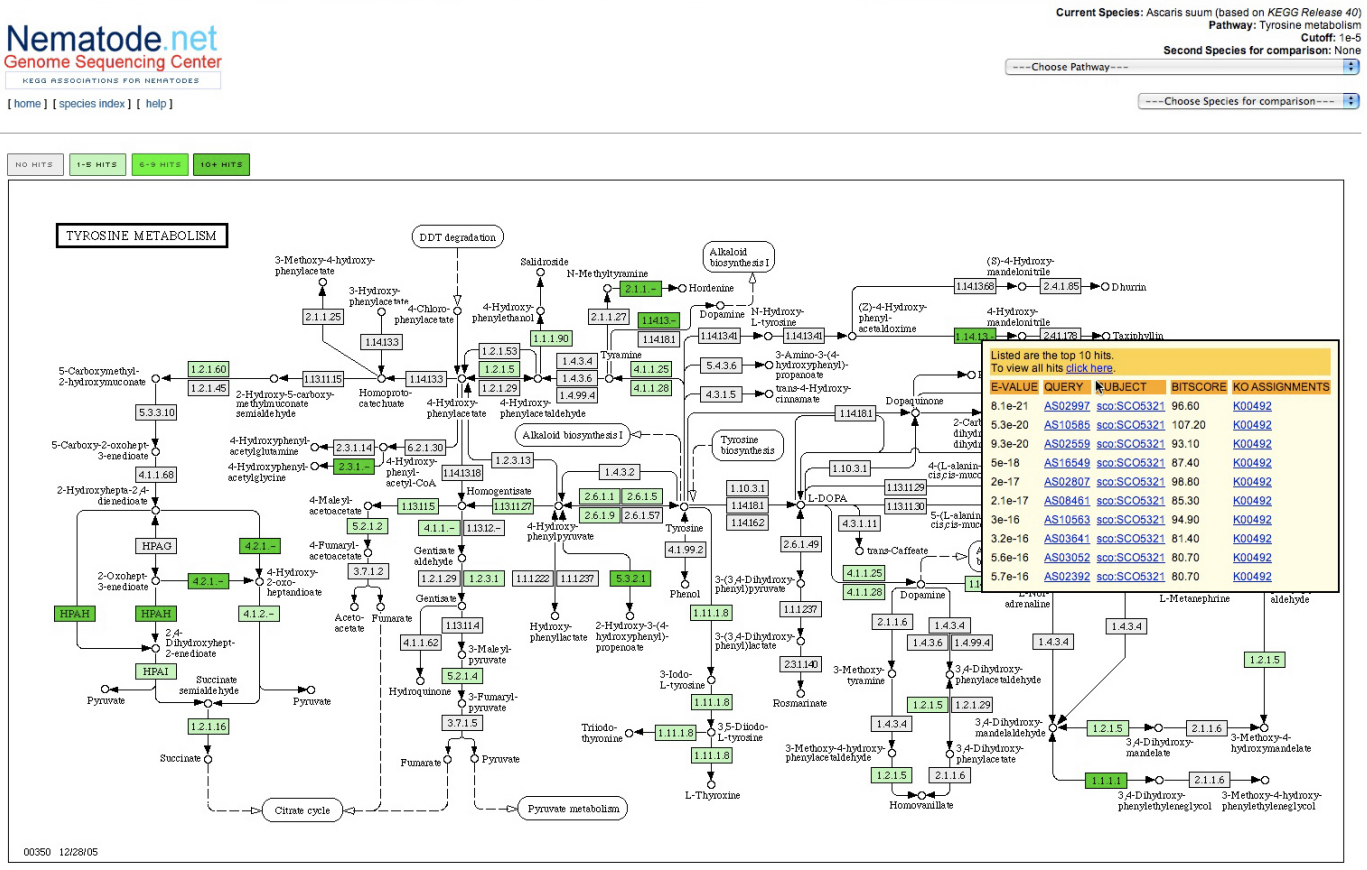
**Pathway Annotation and Summary Hit Table**. The NemaPath pathway viewer showing all of the associated EC id nodes at a specified E-value threshold of 1 × 10^-5 ^or better in the Tyrosine Metabolism pathway for the nematode species *Ascaris suum*. A node with an EC number of 1.14.13.- has been moused-over to reveal a summary hit table for the node, supplying links to more detailed information on the corresponding *A. suum *sequences.

Once an E-value threshold is chosen, a user is presented with a table of pathways available for the chosen species; only pathways with matches based on the threshold the user has selected are presented. Choosing a pathway will present an annotated KEGG pathway image map wherein EC number nodes will be highlighted (colored) based upon number of corresponding matches to the node (darker colors represent enzymes with more matches over those of lighter color); the highlighted nodes are interactive in that a user may position their mouse pointer over the node to reveal a hit summary table (see Figure 2). The table is truncated to the top ten matches based on E-value for brevity – however, a link is provided within the pop-up to a page providing all matches for the node. Each node's hit table provides E-value, subject identifier, corresponding KO numbers, bit score, a link to the EC's summary page on KEGG's web site, and a link to the full NemaGene parent cluster page for the query contig sequence. In turn, on the NemaGene cluster page, displayed in GBrowse, there is a link to the pathway in which the corresponding putative enzyme encoded by the query is active. Pathway nodes that do not have matches link directly back to the KEGG entry for the node's EC number entry. Additionally, on the cluster page for each EST contig the counterpart *C. elegans *entry is listed and a link provided to the corresponding elegans gene page in WormBase.

**Figure 3:**
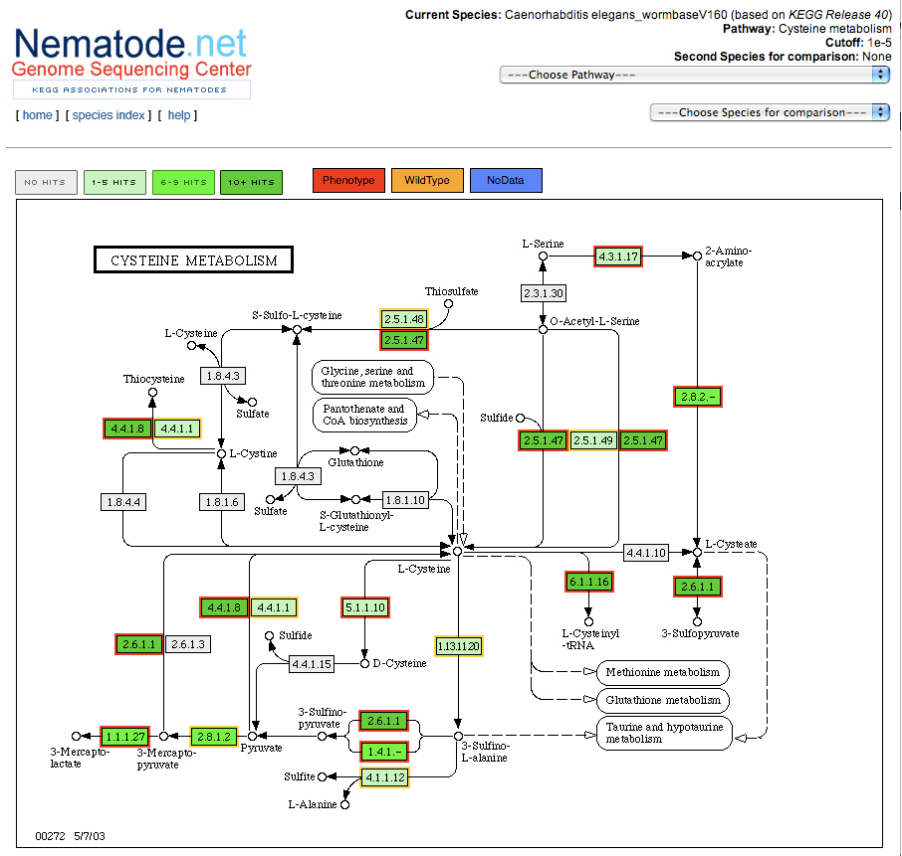
**RNAi Phenotype Association in *C. elegans***. The NemaPath pathway viewer displaying RNAi phenotype information for C. elegans mappings to the Cysteine metabolism pathway at the specified E-value of 1 × 10^-5 ^or better. Nodes outlined in red indicate a mapping between that EC id and a *C. elegans *gene with a known RNAi knockdown phenotype. Nodes with a yellow border indicate a mapping to a *C. elegans *gene where RNAi knockdown resulted in the wildtype trait. Blue borders (none shown here) would indicate that a *C. elegans *gene for which no RNAi information is known is mapping to a given EC id node.

In addition to EST sequence information for multiple species, NemaPath also provides association for four nematode species (*C. elegans*, *B. malayi*, *A. suum *and *A. caninum*) for which complete or partial gene sets are available based on finished or low coverage genome sequence. The *C. elegans *associations are supplemented with the information on RNA interference (RNAi) knockdown [23] candidates. RNA interference (RNAi) has become an efficient high-throughput approach for rapidly determining the phenotypic effects of transcript knockdown in many organisms, including *C. elegans *[24-27]. Gene functions derived from RNAi phenotypes in *C. elegans *can be further extrapolated, to an extent, to orthologous genes in other nematodes where high-throughput screening is not yet practical [28]. Such functions may also help in identifying or confirming chokepoint reactions as potential drug targets [29,30] by uniquely consuming a specific substrate or uniquely producing a specific product in the metabolic network of a species. Therefore, we incorporated RNAi results in our *C. elegans *associations by labeling enzymes within pathways (example in Figure 3) as having associated RNAi phenotypes or not: the enzymes that are "knocked down" after RNAi are colored in red vs. the wild type or no RNAi information (yellow and blue respectively). Chokepoint analyses are particularly straightforward to perform with a computational representation of metabolism, due to the constraints placed on the representation, but would be difficult to perform on a flat list of enzymatic reactions, considering synonyms for reactions and compounds [30].

### Multi-Species Comparison

**Figure 4:**
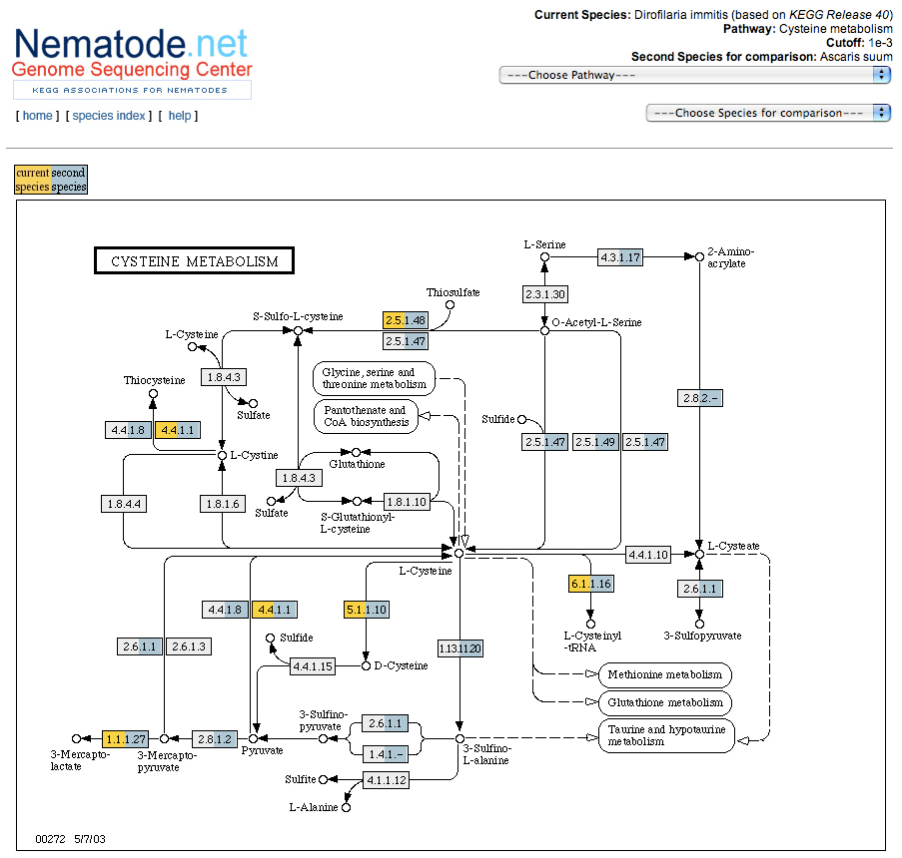
**Multi-Species Pathway Comparison View**. The NemaPath pathway viewer displaying the Cysteine metabolism pathway at a specified E-value of 1 × 10^-5 ^or better for a two species comparison. EC id nodes whose left half are colored in gold indicate a mapping to the primary species selection, in this example *Dirofilaria immitis*. EC id nodes whose right half are colored in green indicate a mapping to the secondary species selection, the example here being *Ascaris suum*.

NemaPath includes a tool to make comparisons between two nematode species on a pathway level based on associations to a given EC id (see Figure 4). Selection of a second species will highlight EC ids with association to either of the two species; a pop-up table provides the top ten matches of highest sequence similarity to the EC ids from both species at the required E-value threshold. This feature facilitates visual identification of differences in EC annotations between the two species. In addition to aiding recognition of different reaction routes in a pathway, enzymes lacking in one of the species or enzymes present in both species may be distinguished.

Other multi-species views include clade-specific and host-specific aggregations that incorporate only the best matches (by lowest E-value better than 1e^-05^) to each gene belonging to a clade or host category. Sequence data are currently available for four (I, III, IV and V) of the five nematode phylogenetic clades. All five clades include parasitic species and parasitism is hypothesized to have arisen independently multiple times [31]. Comparative analyses between these clades may provide valuable information; hence, we provide an interface for viewing clade-specific NemaPath annotations. In addition to clade-based comparisons, we provide a host-specific comparative view that differentiates animal parasites, plant parasites and free-living nematodes.

## Utility and Discussion

### NemaPath Research Application for Nematology

With algorithm improvements and ever-faster expansion of biological sequence databases, sequence comparison has become a basic but critical tool in the post-genomic era. Putative functions of newly obtained sequencing data can be easily inferred by similarity search to the currently characterized proteins when those sequences contain similarities significant enough to be detected on the primary sequence level. However, while individual mappings are frequently used for detailed studies, organized hierarchical annotations of genes provide an understanding of biological systems as a whole – namely how individual genes interact as parts of complexes, pathways, and networks. As a result, nematologists have used the KEGG interaction and reaction network associations for intra- and inter-specific comparative studies and pan-phylum comparative studies. Such *in silico *comparative metabolomics have identified metabolic features that are taxonomically restricted and/or enriched in specific stages or tissues. For example, a pan-phylum analysis based on 93,000 genes of partial genomes in 32 nematode species when compared to the KEGG database identified taxonomically restricted biochemical pathways that may serve to direct drug target definition [32]. Comparative metabolic pathway analysis in the human intestinal nematode parasite *Strongyloides stercoralis *when compared to the metabolic pathways of the free-living nematode *C. elegans*, revealed down regulation of nucleotide sugar metabolism in infective L3 and *dauer *stage which is consistent with the lack of new cell division and DNA replication in these developmentally arrested stages [14]. Dissimilar expression profiles of genes for metabolic enzymes of *Heterodera glycines *infective J2 and *C. elegans *have been reported [33] based on in-depth analysis of ESTs and microarray expression data. Finally, comparison of relative coverage of metabolic enzymes of the adult heartworm *Dirofilaria immitis *compared to *C. elegans*, have supported a hypothesis that the adult heartworm *D. immitis *takes advantages of a anaerobic electron transfer-based energy generation system distinct from the aerobic pathway utilized by its mammalian hosts [34], an observation leading to a promising candidate pathway for development of new macrofilaricides. A hypergeometric statistic test on the extent of KEGG Orthology (KO) groups in the first tissue-level comparative study of nematode intestines has revealed that the major pathways of carbohydrate metabolism and energy metabolism are two commonly over-represented metabolic features in intestines of gastrointestinal parasitic nematodes *Ascaris suum*, *Haemonchus contortus*, and the free-living *C. elegans *[35].

As a step towards a full-scale genome project of *Ancylostoma caninum *– a hookworm of canids used as a model for studying human infections – 104,000 GSSs were generated and subsequently assembled into 57.6 Mb of unique sequence, resulting in gene identification of 9113 non-redundant genes (5538 based on GSSs and 3575 based on ESTs); functional classifications of many of the 70% of genes with homology to genes in other species were possible based on gene ontology and KEGG placement [36].

### Validations and Limitations

Every effort has been made to improve the quality and fidelity of EST sequences by NemaGene clustering. However, final cluster products are putative, partial gene representations built on the most current information available. Enzyme Commission numbers are assigned to NemaGene contigs in the NemaPath database by primary sequence similarity. As such, associations include the caveats pertaining to any automated, high throughput alignment analysis.

To assess the accuracy of our methods, the NemaPath associations of the complete full-length gene set for *C. elegans *was compared to the metabolic pathways in *C. elegans *in the KEGG database. This validation screen was performed using two cutoffs (1e^-30 ^and 1e^-100^) and identified a number of unique EC ids by the original KEGG associations and also our NemaPath associations. Using 1e^-30 ^and 1e^-100 ^as a cutoff, we identified that 1e^-100 ^gave a number of unique EC mappings closer to the original KEGG associations. Only in 4 out of 94 pathways were the number of EC ids identified by NemaPath were lower than in KEGG's *C. elegans *reference pathway. The revealed differences, consistently higher EC ids identified by NemaPath, are mainly based on the manual curation of the KEGG mappings compared to our automated associations guided by cutoff value; our associations do not account for the ancillary information included in KEGG Orthology numbers (KO) generation.

KO numbers incorporate ancillary information (i.e., manual curation) that is not represented in the EC number annotation. KO is a further extension of this scheme (similarity-based automatic EC id association) based on computational analysis – as well as manual curation – of SSDB ortholog clusters in order to classify all gene functions and explore unknown pathways [37]. NemaPath does provide direct representation of KO assignment, but does not exclude EC associations in specific pathways based on *a priori *KO knowledge. By design, an association of a user's query sequence to a particular EC identifier will be highlighted in every pathway where the EC id exists, rather than exclude information from the end-user or enforce presumptive omissions. Partial EC numbers (e.g., 1.1.-.-) are not excluded; care must be taken in their interpretation. The ambiguous nature of the partial EC number allows different enzymes that catalyze different reactions to share the same identifier within the same class, even though this does not necessarily mean they have the same activities [38].

Because we do not provide exhaustive curation, proper interpretation requires the user's cognizance of the metabolic pathways, as well as an understanding of KEGG annotation and vocabulary.

### Future Directions

All species-to-KEGG associations in NemaPath are re-compiled each time KEGG releases a new version of their genes database (as of this writing the associations are made using KEGG release 46). New nematode species are added post NemaGene clustering. Genome sequencing projects of several free-living and parasitic nematode species are underway or planned [39], and the associations to KEGG metabolic pathways will be included as the data become available. Furthermore, future builds of NemaPath will not be limited to metabolic pathways, nematode sequence associations will include other manually drawn pathway maps representing molecular interactions and reaction networks. Also, further development will allow multi-species comparison beyond the current two-species similarity view supported by NemaPath.

## Conclusion

NemaPath, the database described herein, provides the research community a unique resource for pathway visualization of multi-species nematode genomic data in terms of KEGG database vocabulary. NemaPath is part of the larger Nematode.net web resource and integrates well with the site's previous functionality, streamlining access to significant internal nematode sequence data, as well as information provided by off-site resources at NEMBASE3 [40] and WormBase [41] repositories.

## Availability and Requirements

For accessing the Nematode.net web site, direct your web browser to  on the World Wide Web. Direct access to the NemaPath pathway browser – as well as species-specific tree-views – can be found at  URL. Plaintext file versions of alignment information (as compiled by KEGGscan) are also available by species . The most current NemaGene EST cluster builds are available via FTP at  after completing a short access form. Please contact the authors concerning access to ancillary information associated with this resource.

## Authors' contributions

TW developed the software and wrote the draft manuscript. JM, SA, and DM helped develop software and informatics approaches for the Nematode.net web site. YY and ZW contributed pathway analyses for specific nematode sets. JPM and MM initiated the parasitic nematode project at Washington University School of Medicine, St. Louis. All authors contributed to the writing and editing of this manuscript.
